# Selection of optimal strategy for managing decentralized solar PV systems considering uncertain weather conditions

**DOI:** 10.1038/s41598-024-62891-6

**Published:** 2024-05-28

**Authors:** Assia Chadly, Hamed Yahya Aldayyani, Mohammad M. Hamasha, Sa’ed Amer, Maher Maalouf, Ahmad Mayyas

**Affiliations:** 1 Department of Engineering Systems & Management, Khalifa University, Abu Dhabi, UAE; 2 Department of Industrial Engineering, Faculty of Engineering, The Hashemite University, Zarqa, 13133 Jordan

**Keywords:** Solar PVs cleaning, MCDM, AHP, QFD, FTOPSIS, PSI, Energy science and technology, Energy harvesting, Renewable energy

## Abstract

Solar power is a promising source of energy that is environmentally friendly, sustainable, and renewable. Solar photovoltaic (PV) panels are the most common and mature technology used to harness solar energy. Unfortunately, these panels are prone to dust accumulation, which can have a significant impact on their efficiency. To maintain their effectiveness, solar photovoltaics s must be cleaned regularly. Eight main techniques are used to clean solar panels: natural, manual, mechanical, robotic, drone, coating, electrical, and acoustic. This study aims to identify the best cleaning method using multiple criteria decision-making (MCDM) techniques. Using the Analytical Hierarchy Process (AHP), Quality Function Deployment (QFD), Fuzzy Technique for Order of Preference by Similarities to Ideal Solution (FTOPSIS), and Preference Selection Index (PSI), this research evaluates all eight cleaning methods based on several criteria that are categorized under cost, performance, resource requirement, and safety in Abu Dhabi. The data are collected from surveys completed by experts in solar and sustainable energy. The AHP, QFD, and PSI results identified natural, manual, and surface coating as the best and most effective cleaning methods. Natural cleaning involves using rainwater primarily to remove dirt and dust; manual cleaning requires cleaning agents and wiping clothes; and surface coatings involve applying a layer of hydrophobic material to the panels to repel dust. Identifying the most effective cleaning method for dust removal from solar panels can ensure optimal efficiency recovery at minimal costs and resources.

## Introduction

**Figure 1:**
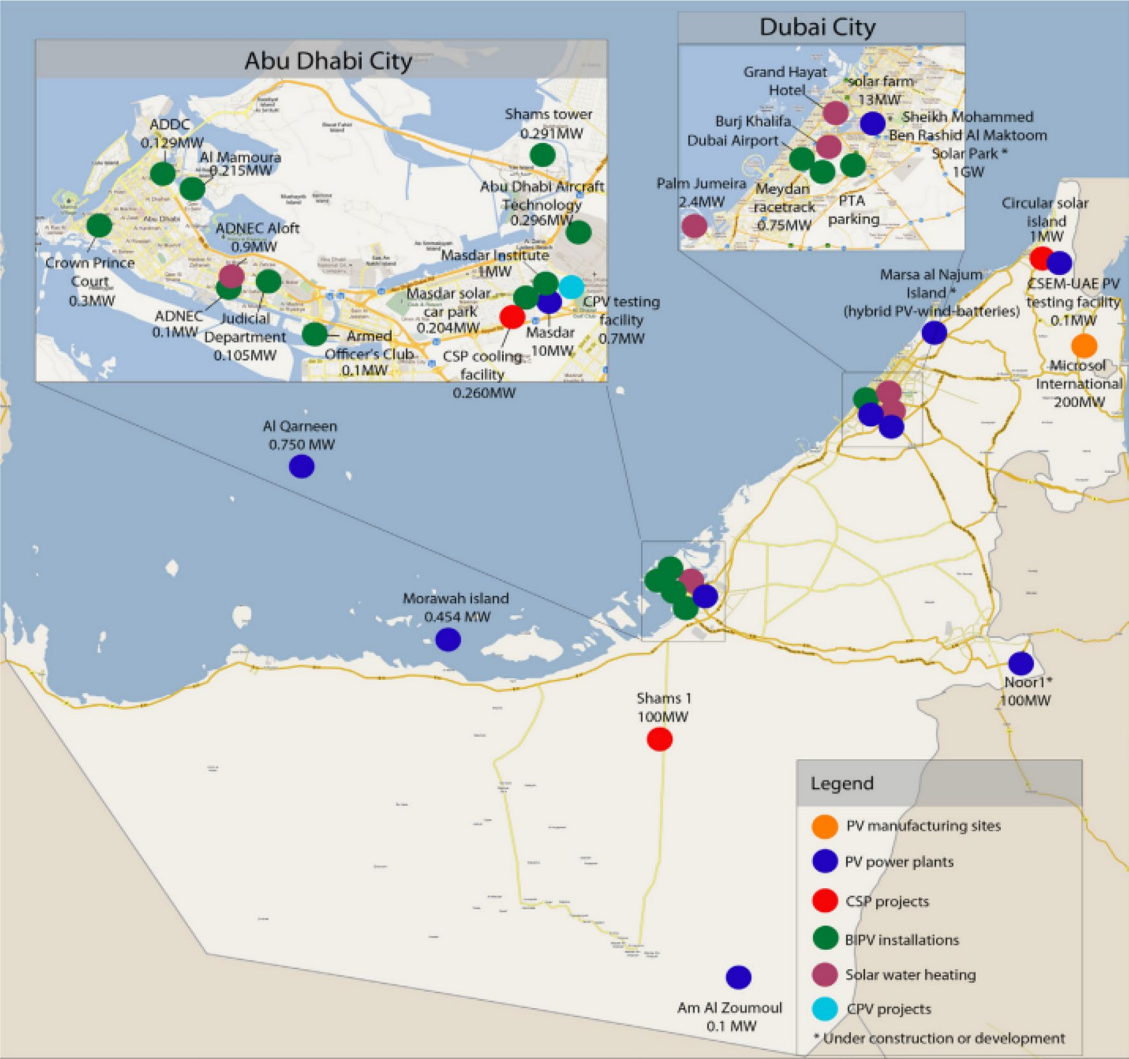
Map of the solar installations in the UAE^7^.

Increases in the price of fossil fuels, concerns about air quality and human health, and the environmental impact of fossil fuel dependence have all contributed to the switch to renewable energy sources such as solar power^1^. Photovoltaic (PV) cell arrays are the most common way to harness solar energy, and PV electricity is expected to offer more than a 20% share of global energy consumption by 2050^2,3^. However, the maintenance of these solar PVs is a challenge due to the soiling and dust accumulation on the panels. That causes lower energy conversion efficiency because the solar cells are blocked. Dust-saturated environments could suffer a loss of more than 50% in generated PV power due to soiling^4–6^. Due to dust, the amount of solar irradiation reaching the cells is reduced, and the cell temperature increases. The effects of soiling and dust accumulation vary from one location to another due to factors such as the source of dust particles, the particle type, the particle’s physical and chemical properties, the wind velocity, the humidity and rainfall, the PV module technology used, and the material of PV module surface cover^7,8^. Regions with rainfall scarcity are prone to high levels of dust accumulation on PV panels; for example, countries in the Arab peninsula are characterized by hot and humid environments that experience significant reductions in power output^8^. Therefore, periodic solar panel cleaning is extremely important to maintain the performance and highest power output of PV modules^9^. Mokry et al.^7^ summarized the main solar projects in the UAE up to 2013 and mapped them as shown in Fig. 1. Several solar projects, such as the Mohammed bin Rashed Al Maktoum Solar Park and Al Dhafra Solar PV project, and programs like the Dubai Clean Energy Strategy 2050 were initiated shortly after that^10^.

Different cleaning methods and technologies have been implemented to overcome this challenge, such as natural cleaning, manual cleaning, mechanical cleaning (sprinkler system), robotic cleaning, and surface coatings^11^. Other advanced methods include drone retrofitting, electrostatic cleaning, and ultrasonic cleaning; these are currently in the research stage but show promising results. The features and drawbacks of this cleaning method influence its choice. Therefore, selecting the best cleaning method is more complex due to several factors including economic feasibility^5^. Failure to implement the appropriate method could result in significant financial losses that could adversely impact the levelized cost of energy of PV modules, thus reducing the desire to invest in such technology^12–14^. Similarly, opting for cheaper alternatives could result in inadequate recovery of lost power output due to poor cleaning capability. Therefore, to evaluate and select the most optimal cleaning method, it is necessary to consider capital cost, operational & maintenance cost (O&M cost), cleaning efficiency, cleaning time, water consumption, electricity expenditure, and safety.

Farrokhi et al.^15^ stated that most countries with the capacity to utilize solar energy via photovoltaic (PV) panels suffer from dust accumulation, and the removal of surface impurities is required to improve the efficiency of solar panels. Khadhka et al.^11^ concluded that soil concerns may limit the energy harvesting potential of solar power plants, as dust collection on PV panels can impair the system’s efficiency and power output by up to 80%. Said et al.^8^ summarized the effect of dust on PV performance in some countries around the world and concluded that the degradation of the efficiency went beyond 15% in a period that does not exceed three months. They showed that the degradation rate reached 5% in Palestine in one week, 7% in Saudi Arabia 17.4% in Egypt in one month, 10% in the UAE in five weeks, 6.5% in Athens in six weeks, 15% in Spain and 11.5% in the United States in two months, and finally 10% in Qatar after 100 days^8^.

Due to the heavy reliance on daily sunshine, dust accumulation hinders the module’s reliability by preventing solar radiation from reaching the surface, which could cause uncertainty in energy production^16^. Consequently, proper cleaning measures must be implemented to avoid system degradation and financial losses. Appropriate cleaning methods are merely based on various parameters that take into account technical and economic standpoints^17^. Based on that, the authors systematically compared the different cleaning methods, such as natural cleaning, manual cleaning, mechanical cleaning, electrical cleaning, ultrasonic cleaning, and surface coatings. Table 1 summarizes the current cleaning methods found in the literature, their working mechanism, advantages, disadvantages, estimated cost level, and efficiency improvement of each method. This comparison should help by providing direction when selecting a suitable cleaning technique under specific settings such as the plant scale, environmental condition, and budget. It should be emphasized that the efficiency data was directly extracted from the original articles and are meant for comparison purposes. However, it cannot be used to draw a conclusive judgment concerning the superiority of one cleaning technique over the other. The results relied heavily on several variables and factors, such as the cleaning frequency, the amount of soil on the panel surface, and the study period. However, it can provide a hint about the cleaning capacity of some methods. In addition, it is important to note that the cost levels are not based on factual data but rather averaged according to the limited information in the literature.

**Table 1:** List of different cleaning methods found in the literature.

Category	Cleaning Tech/Method	Mechanism	Pros	Cons	Estimated Cost*	Efficiency Improvement**
Natural Cleaning ^4,8,14,18,19^	Rainfall Wind Gravity Tilted Panel	- Rain washes away dust particles - Wind blows away dust - Tilted panels force particles to fall due to gravity	- No costs - Frequent rainfall could maintain panel efficiency	- Unreliable in dry regions - Depends on weather - Could worsen soiling through mud formation	N/A	Unpredictable
Manual Cleaning ^2,3,11,15^	Labor Cleaning kits Detergents Water Cloth	- Manpower utilizes different tools, such as brushes and wipers, to clean the panels - A mixture of water & detergents can be used	- Very effective cleaning - Good choice for small scale	- Inefficient for large scale - Human risk factor - Not feasible for remote locations - Requires water source	Low	Up to 98%
Mechanical Cleaning ^5,18,20–23^	Heliotex sprinkler system	- Consist of a water supply system - Attaches nozzles to solar panels that spray water all over - Uses switch timer to activate	- Water reaches the entire surface - Can reduce surface temperature - Requires less effort	- High water consumption - Requires maintenance	Moderate	Up to 3%
	PIC & PLC-based cleaning	- A water system is used to wet the panels, and a flat wiper is used for cleaning - Rotating roller blades are used to clean the panel	- Automated - Daily cleaning - Low maintenance cost	- High water consumption - Moving parts - High initial cost (PLC)	Moderate	No available data
Electrical Cleaning ^6,15,16,24^	Electrodynamic screens	- High voltage is used to charge particles, charged particles repel each other to the edge of the panel	- Very efficient cleaning - No water required - Good for desert regions	- High initial cost - Less effective for fine dust particles - Degradation of plastic screen due to UV	High	Up to 90%
Robotic Cleaning ^25–29^	Robotic dry cleaning	- Robot utilizes silicon rubber foam brush	- Waterless - Does not damage the panel surface - Automated - Suitable for frequent cleaning	- Heavy - Installed (not portable) - Not effective against wet particles	Low	No available data
	Robotic wet cleaning	- Robot utilizes wipers, brushes, and water sprayer	- Long life expectancy - Fast cleaning rate - Can remove persistent dirt	- Requires a person to operate - Low panel inclination - Expensive	High	No available data
	Arduino Due Robot	- Robot operates without rails or guides that wipes dust using helical brushes	- Waterless - Autonomous - No guide rails required - Self-guided using sensors - Suitable for desert areas	- Requires charging - Requires sun-shaded parking - Slow on tilted panels	Low	No available data
Drone Cleaning ^30^	Drone retrofitting	- Microfiber based-cloth wiper or microfiber & vacuum cleaner combination is retrofitted on an unmanned drone	- Waterless - Autonomous - Mobile - Suitable for large-scale plants - Suitable for desert region	- Short flight time - Requires frequent charging	High	Up to 7.7%
Coating Cleaning ^14,31–36^	Hydrophobic Coating	- PV panel surface is coated with hydrophobic SiO2 nanomaterial	- Passive and does not require a power source - Anti-reflective (reduces panel temperature)	- Requires water to roll dust off - Coating degrades over time	Moderate	Up to 15%
	Hydrophilic Coating	- Diffusing water on TiO2 film-coated surface, which rinses dust	- Passive and does not require a power source - Reduce dust deposition	- Water is required	Moderate	No available data
Acoustic Cleaning ^37^	Ultrasonic Cleaning System	- Utilizes cavitation effect in thin water layer for cleaning	- Able to infiltrate hard-to-reach surfaces - Integrated on solar panel (good option for desert regions)	- A thick water layer is required - Efficiency depends on several parameters	High	No available data

*Estimated costs are assumed and are not based on actual data.

**Efficiency data was directly extracted from original articles.

Gupta et al. conducted a comprehensive review study on the effect of dust on solar PV and potential mitigation techniques^6^. Based on current and historical research, they concluded that selecting the best dust-cleaning approach must include natural and artificial mitigation techniques^6^. Younis and Onsa^4^ summarized the cleaning operations most frequently used in the regions of Africa and the Middle East and their impact on PV performance. Their analysis shows that different climates require different cleaning methods. For example, surface coating is more suitable for African countries, while water-based cleaning is more efficient in the Middle East. They also concluded that natural cleaning is ineffective in areas with a dry climate. Knowing which cleaning method suits any given region should also consider its challenges and potential opportunities. As such, an improvement of around 25% in power output can be achieved if proper cleaning mechanisms are used^5^. That calls for the development and improvement of automated cleaning methods in dry regions that suffer from water scarcity and the research of possible developments in cleaning processes such as electrical, mechanical, and chemical. In arid regions, manual tools and installed hydraulic systems are the optimal methods based on cost, labor, and water usage^38^. The same authors addressed the need for commercially feasible cleaning solutions and introduced candidate technologies that could demonstrate great potential in preserving solar energy harvesting in sun-rich desert regions^38^.

Ultimately, given the different cleaning alternatives and several criteria, only a Multi-Criteria Decision Making (MCDM) process could provide the optimal cleaning solution for each situation. MCDM is a process that works by finding the optimal solution or alternative from a set of two or more choices concerning the established conflicting criteria upon which the solutions are evaluated. It is applied to different fields to obtain an optimum solution to a problem where there are many parameters to consider that cannot be decided by user experiences. To help the decision-maker choose an alternative with the fewest trade-offs and the most benefits, it analyzes and compares criteria against every other choice. Both qualitative and quantitative criteria can be employed in the analysis. The application returns a ranking result based on the chosen criteria, their matching values, and the specified weights.

There are various methods for solving MCDM problems: Preference Selection Index (PSI), Analytical Hierarchy Process (AHP), Analytical Network Process (ANP), Quality Function Deployment (QFD), Elimination and Choice Expressing Reality (ELECTRE), Technique for Order of Preference by Similarities to Ideal Solution (TOPSIS), Fuzzy TOPSIS (FTOPSIS), and grey theory are a few examples. For our research, we chose AHP, QFD, FTOPSIS, and PSI to rank the different cleaning methods and select the best alternative according to the set of selected criteria that cover the economic, technical, and applicability of the cleaning methods in any given geographical location. AHP is a tool that uses simple mathematical models to distinguish between different options when multiple interrelated objectives must be achieved. AHP is the most used MCDM method in the literature^39^. QFD is a customer-oriented approach that usually begins by gathering and integrating customer requirements into the product design. TOPSIS is based on a clear set of criteria and mathematical calculations considering both positive and negative attributes of alternatives. It is well-suited for decision-making situations where trade-offs between different attributes are necessary. Fuzzy TOPSIS considers degrees of truth, allowing truth values to range from 0 to 1. This approach is useful for handling real-world situations where the information used to make decisions is unclear, vague, or conflicting. PSI transforms subjective preferences conveyed by decision-makers into a numerical scale, providing a more thorough and objective basis for evaluating alternatives. By assigning preference strengths to different choices, PSI helps to compare and rank alternatives based on multiple criteria. It allows for aggregating individual preferences, consistency assessment, and sensitivity analysis. It ultimately improves the transparency and quality of decision-making in MCDM to reach optimal and reliable choices. AHP stands out as the most frequently utilized MCDM method^39^. PSI, on the other hand, is designed to be bias-free, preventing the over or underestimation of selection criteria. In this study, we have customized QFD to transform it into a unique MCDM method by incorporating experts’ evaluation with technical factors and then mapping technical factors to the project owner’s needs by selecting the best cleaning method for solar panels, while FTOPSIS excels in handling uncertain data instead of just singular values. The application of these four methods enhances the diversity of the analysis and strengthens the decision-making process.

Obeidat^12^ used the PSI approach to select the optimal solar panel cleaning method between manual, heliotex, electrostatic, automatic, and self-cleaning. The criteria were based on six attributes, some of which are cost, performance, and safety. The PSI results suggested that the most suitable cleaning technique was manual cleaning followed by heliox technology, etc. On the other hand, AlMallahi et al.^40^ used the TOPSIS approach to find the optimal dust removal method in solar panels in the UAE between robotic, manual, and nano-coating cleaning techniques based on cost, performance, environmental, and economic impact. Their results showed that the optimal cleaning method is robotic (water-based), followed by manual cleaning, robotic (pressure-based), and nano-coating was the least preferred. Furthermore, Aljaghoub et al.^41^ compared different cleaning techniques concerning the criteria of the Sustainable Development Goals (SDG). According to their results, manual cleaning was the best alternative.

This paper aims to compare the feasibility of all the cleaning techniques listed in Table 1 in the city of Abu Dhabi, UAE. The analysis will consist of information extracted from related published work and surveys that were distributed to experts in the field of solar energy in the city. After that, MCDM models will be utilized to propose an optimal cleaning method for solar panels based on several presumed criteria in terms of cost, performance, resource requirement, and safety. AHP will be used first due to its simplicity and straightforward application. QFD will be implemented to take into account the user’s needs when deciding on the different alternatives. The fuzzy theory will be combined with the TOPSIS method to account for uncertainty and imprecision in decision-making. Finally, PSI will be implemented to facilitate a more robust and informed decision-making process in complex and uncertain environments.

While the Middle East region is mostly studied for dust accumulation in solar panels, no article compares all the possible cleaning methods in a single city/country. In addition, we are relying on the input of experts in the field via surveys rather than personal judgment. Finally, we employ several MCDM techniques to validate our results. This research is novel in the sense that it compares the results of applying four different MCDM techniques to select the most optimal dust-cleaning technique among 4 methods while considering 7 main criteria. In the literature, several research articles focused on one MDCM technique only and fewer cleaning techniques and criteria. This makes our study thorough and holistic as it considers a multitude of variables and assessment techniques. In addition, this study is among the first studies that focus on arid regions while considering weather and technological factors simultaneously. The other important contribution of this study is the comprehensive sensitivity analysis of each method to understand how changes in the most influential criteria affect the final results. The significance of this work pertains to cases in which it is possible to apply certain novel dust-cleaning methods, such as drone and acoustic cleaning. In addition, this study could provide a solid basis for areas of similar weather, desert regions that sometimes experience heavy rain and thunderstorms. Moreover, the research is done about important criteria, some of which are generally disregarded, such as cleaning time and level and water consumption. These may not seem crucial, but their impact can be heavy in other areas.

The rest of the paper is organized as follows: Section “Methodology” will present the methodology followed in this research by defining the selected MCDM techniques, the criteria, and the alternatives. The results, along with their discussion, are presented in Section “Result and discussion”, and the limitations of this study are highlighted in Section “Limitations” before concluding in Sections “Conclusion and future work”.

## Methodology

### Analytical hierarchy process

The Analytical Hierarchy Process (AHP) is a powerful multi-criteria decision-making tool developed by Saaty^42^ for managing qualitative and quantitative multi-criteria elements in decision-making. It is a comprehensive system for making decisions with multiple criteria by structuring the process in a hierarchical manner. It uses pair-wise comparisons to illustrate the compatibility or incompatibility of elements while making a decision. A hierarchy is created, and each level is assigned a value; then, those values are used to form a matrix to compare all the existing elements. The values go from 1 to 9, with 1 indicating that two elements are equally significant and 9 indicating that one element is significantly more important than the other. The pairwise scale and the importance value attributed to each number are illustrated in Table 2.

**Table 2:** Scale for pair-wise comparison.

Intensity of Importance	Definition	Explanation
1	Equal importance	Two activities contribute equally to the objective
3	Moderate importance	Experience and judgment slightly favor one activity over the other
5	Strong importance	Experience and judgment strongly favor one activity over the other
7	Very strong importance	An activity is favored strongly over the other; its dominance demonstrated in practice
9	Extreme importance	The evidence favoring one activity over the another is of the highest possible order of affirmation
2,4,6,8	Intermediate Values	When compromise is needed

To conduct AHP, the following is the algorithm showing the required steps and calculations^43^:

1. Introduce the problem, the objective, the alternatives, and the decision criteria.
2. Create and distribute a survey to gather opinions from experts in the field. The participant should indicate their preferred level of importance for each option; then, the average will form the group’s overall judgment.
3. Create a pairwise comparison matrix for the criteria.
4. Create a normalized pairwise comparison matrix using a scale similar to the one shown in Table 2.
5. Calculate every criterion’s weight.
6. Find the largest eigenvalue and calculate the consistency index and ratio.
7. Create pairwise and normalized pairwise comparison matrices for alternatives with respect to each criterion.
8. Find the weights of each alternative with respect to each criterion.
9. Calculate each alternative’s score.
10. Rank the alternatives based on their scores and select the best alternative as the one with the highest score.

The process of comparing pairs of elements generates a matrix of relative rankings for each level of the hierarchy. The number of matrices created is based on the quantity of elements at each level. The arrangement of the matrix at each level is dictated by the number of elements it is linked to in the level immediately beneath it. Once all matrices have been formed and all pair-wise comparisons have been made, calculations are done to determine the eigenvectors, or relative weights, global weights, and the highest eigenvalue (λ_max_) for each matrix^44^. The λ_max_ value is a crucial factor of AHP; it serves as an index to assess information by determining the Consistency Ratio (CR) of the calculated vector. This ensures that the pair-wise comparison matrix provides a consistent evaluation. The consistency ratio is calculated as follows:

1- Calculate the largest eigenvalue λ_max_ for each matrix of order n.

2- Compute the Consistency Index (CI) for each matrix of order n by the formula:

1 $${CI = \left( {\lambda_{ max} - n} \right)/\left( {n - 1} \right)}$$

3- The consistency ratio is then calculated using the formula:

2 $$CR=CI/RI$$

RI, the Random Index is determined through numerous simulation runs, and changes based on the matrix’s order, are already known. Table 3 shows the values of the RI based on n^44^.

**Table 3:** Average random index (RI) based on matrix size (adapted from Saaty) ^42^.

n	1	2	3	4	5	6	7	8	9
RI	0	0	0.52	0.89	1.11	1.25	1.35	1.40	1.45

The range of CR that is considered acceptable depends on the matrix size, with a lower limit of 0.05 for a 3 × 3 matrix, 0.08 for a 4 × 4 matrix, and 0.1 for larger matrices where $n \ge 5$. If the CR value falls within this range or is lower, it suggests that the evaluation is consistent, and the comparative judgments represented in the matrix are reliable. However, if the CR value exceeds the acceptable limit, it indicates inconsistencies in the decisions, and the evaluation process should be reevaluated and improved^44^.

### Quality function deployment

Quality Function Deployment (QFD) is a systematic method of product and service design that emphasizes meeting customer requirements by analyzing customer needs and using this information to construct a “House of Quality” matrix^45^. The matrix helps prioritize customer needs and identify relationships between them and design characteristics. The design is continuously improved through feedback loops and testing, ensuring a high-quality final product that meets customer expectations^46^.

The House of Quality (HOQ) is a matrix diagram that translates customer requirements into engineering requirements. It is the central tool in QFD and is used to link customer needs to product features, design characteristics, and process capabilities. The HOQ is typically represented as a matrix with customer requirements on one axis and product features or design characteristics on the other axis. The cells in the matrix represent the relationship between customer requirements and product features and can be used to assess the strength of that relationship. This information can then be used to prioritize customer requirements and determine which product features are most important to customers^45,46^. The HOQ also helps identify technical trade-offs that must be made in product design^46^.

The steps to create a HOQ are as follows^46^:

1. Specify the customer needs and the engineering requirements as the “what” and “how” windows, respectively.
2. Define the weights from 1 to 10, where 1 is the least important and 10 is the most important.
3. Define the relationship scores between the customer needs and the engineering requirements between 1 and 3, where 1 is weak, 2 is medium, and 3 is strong.
4. Define the interrelationship scores between the engineering metrics from 1 to 3, where 1 represents an inversely proportional relationship, 2 means no relationship, and 3 represents a directly proportional relationship.
5. Indicate the direction of the improvement, upward or downward.
6. Calculate the targets, which are:
  - The raw score is the sum of the product of the weights of the customer’s needs and engineering requirements.
  - The relative weight: the weight of the normalized score divided by the sum of all the weights.
  - The rank: the descending ranking of the engineering metrics with respect to their relative weights.
  - The technical requirements targets: the product of the interrelationship scores and the relative weights.
  - The technical rank is the descending ranking of the engineering metrics with respect to the technical requirements target.

The resulting relative weights will then be used against the alternatives, and the raw score, relative weight, and rank are computed again to conclude which alternative is the most optimal.

### Fuzzy technique for order of preference by similarity to ideal solution

Fuzzy logic is a mathematical method that enables reasoning with approximate values rather than exact numbers. It has been widely used in various fields, such as control theory, engineering analysis, artificial intelligence, medical analysis, etc. Fuzzy logic is different from traditional binary sets, which only allow for true (1) or false (0) values, as it allows for a range of truth values between 0 and 1, meaning that it can deal with the concept of partial truth^47^. Using FTOPSIS, the alternative that is closest to the Fuzzy Positive Ideal Solution (FPIS) and farthest from the Fuzzy Negative Ideal Solution (FNIS) is considered the optimal alternative^48^. In our research, we consider the triangular fuzzy TOPSIS, where attributes are represented by a triplet $(a,b,c)$ that are real numbers. These represent fuzzy ratings for each assessment, as shown in Table 4^48^:

**Table 4:** Fuzzy numbers and ratings corresponding to different qualitative ratings.

Assessment	Qualitative Rating	Fuzzy Number
Very Poor	Very Low	(1,1,3)
Poor	Low	(1,3,5)
Fair	Medium	(3,5,7)
Good	High	(5,7,9)
Very Good	Very High	(7,9,9)

The steps to conduct an FTOSIS analysis are the following^48^:

1. Identify the alternatives $\left({A}_{i}\right)$ and the criteria $\left({C}_{j}\right)$ where $i=\text{1,2},\dots ., n; j=\text{1,2},\dots \dots .,m$.
2. Calculate the aggregated fuzzy weight ${(\widetilde{w}}_{j})$ of each criterion using the equation below:
  3 $$\left.\begin{array}{c}{\widetilde{w}}_{j}=\left({a{\prime}}_{j},{b{\prime}}_{j},{c{\prime}}_{j}\right) and\\ {a{\prime}}_{j}=\text{min}\left\{{{a}{\prime}}_{j}^{k}\right\}; {b{\prime}}_{j}=\frac{1}{K}\sum_{k=1}^{K}{b{\prime}}_{j}^{k}; {c{\prime}}_{j}=\underset{k}{\text{max}}{c{\prime}}_{j}^{k}\end{array}\right\}$$
  where $\left({a{\prime}}_{j},{b{\prime}}_{j},{c{\prime}}_{j}\right)$ are the triplet that contribute to the fuzzy weight ${\widetilde{w}}_{j}$ of each criterion $j$.
3. Calculate the aggregated fuzzy rating ${(\widetilde{x}}_{ij})$ of each alternative under each criterion using:
  4 $$\left.\begin{array}{c}{\widetilde{x}}_{ij}=\left({a}_{ij},{b}_{ij},{c}_{ij}\right) and\\ {a}_{ij}=\text{min}\left\{{a}_{ij}^{k}\right\} ; {b}_{ij}=\frac{1}{K}\sum_{k=1}^{K}{b}_{ij}^{k}; {c}_{ij}=\underset{k}{\text{max}}{c}_{ij}^{k} \end{array}\right\}$$
  where $\left({a}_{ij},{b}_{ij},{c}_{ij}\right)$ are the triplet that contribute to the fuzzy rating ${\widetilde{x}}_{ij}$ of alternative $i$ with respect to criterion $j$.
4. Construct the fuzzy decision matrix and the normalized fuzzy decision matrix using:
  5 $$\widetilde{R}={\left[{\widetilde{r}}_{ij}\right]}_{n\times m}$$
  where in case the decision-maker aims to be closer to the FPIS:
  6 $$\left.\begin{array}{c}{\widetilde{r}}_{ij}=\left(\frac{{a}_{ij}}{{c}_{j}^{*}},\frac{{b}_{ij}}{{c}_{j}^{*}},\frac{{c}_{ij}}{{c}_{j}^{*}}\right)and\\ {c}_{j}^{*}=\text{max}{c}_{ij}\end{array}\right\}$$
  Or, in case the decision-maker targets getting farther to the FNIS:
  7 $$\left.\begin{array}{c}{\widetilde{r}}_{ij}=\left(\frac{{a}_{j}^{-}}{{c}_{ij}},\frac{{a}_{j}^{-}}{{b}_{ij}},\frac{{a}_{j}^{-}}{{a}_{ij}}\right)and\\ {a}_{j}^{-}=\text{min}{a}_{ij}\end{array}\right\}$$
5. Construct the weighted normalized fuzzy decision matrix using the equation below:
  8 $$\widetilde{V}{=[{\widetilde{v}}_{ij}]}_{n\times m}=\left(\begin{array}{cccc}{r}_{11}{w}_{1}& {r}_{12}{w}_{2}& \dots .& {r}_{1m}{w}_{m}\\ {r}_{21}{w}_{1}& {r}_{22}{w}_{2}& \dots & {r}_{2m}{w}_{m}\\ \dots & \dots & {r}_{ij}{w}_{j}& \dots \\ {r}_{n1}{w}_{1}& {r}_{n2}{w}_{2}& \dots & {r}_{nm}{w}_{m}\end{array}\right)$$
6. Determine FPIS ${(A}^{*})$ and calculate the distance ${(d}_{i}^{*})$ of each alternative from it using:
  9 $${A}^{*}=({\widetilde{v}}_{1}^{*}, {\widetilde{v}}_{2}^{*}, \dots ,{\widetilde{v}}_{m}^{*})$$
  where ${\widetilde{v}}_{j}^{*}=(c,c,c)$ such that $c=max\left\{{c}_{ij}^{{\prime}{\prime}}\right\},$
  10 $${d}_{i}^{*}=\sum_{j=1}^{n}{d}_{v}\left({\widetilde{v}}_{ij};{v}_{j}^{*}\right)$$
7. Determine FNIS ${(A}^{-})$ and calculate the distance ${(d}_{i}^{-})$ of each alternative from it using:
  11 $${A}^{-}=\left({\widetilde{v}}_{1}^{-}, {\widetilde{v}}_{2}^{-}, \dots ,{\widetilde{v}}_{m}^{-}\right)$$
  where ${\widetilde{v}}_{j}^{-}=(a,a,a)$ such that $a=min\left\{{a}_{ij}^{{\prime}{\prime}}\right\},$
  12 $${d}_{i}^{-}=\sum_{j=1}^{n}{d}_{v}\left({\widetilde{v}}_{ij};{v}_{j}^{-}\right)$$
  where, ${d}_{v}(\widetilde{a},\widetilde{b})$ is the distance measurement between two fuzzy number $\widetilde{a }({a}_{a},{b}_{a},{c}_{a})$ and $\widetilde{b}{(a}_{b},{b}_{b},{c}_{b})$, calculated as an Euclidean distance:
  13 $${d}_{v}(\widetilde{a},\widetilde{b})=\sqrt{\frac{1}{3}*({\left({a}_{a}-{a}_{b}\right)}^{2}+{\left({b}_{a}-{b}_{b}\right)}^{2}+{\left({c}_{a}-{c}_{b}\right)}^{2})}$$
8. Calculate the Closeness Coefficient $({CC}_{i})$ of each alternative using the equation:
  14 $${CC}_{i}=\frac{{d}_{i}^{-}}{{d}_{i}^{-}+{d}_{i}^{*}}$$
9. Rank the alternatives based on the CC, where the best alternative is the one with the highest CC. That should be the alternative that is closest to the FPIS and farthest from the FNIS.

### Preference selection index

The preference selection index (PSI) method is a simple, direct, and efficient approach to multi-criteria decision-making that involves fewer calculations than other MCDM techniques as it does not require the weighting of criteria and instead relies on statistical concepts. The approach comprises defining the problem, creating a matrix of alternatives and criteria, normalizing the matrix, calculating the preference variation value, determining the overall preference value, calculating the preference selection index, and ranking the alternatives according to ascending or descending order to facilitate the interpretation of the results^49^. This method is distinguished by calculating the PSI value and considering the alternative with the highest value as the best one instead of assigning an importance level to the criteria. However, this decision-making process is associated with a particular belief or expectation of the decision-maker. In other words, the final decision depends on the decision-maker’s belief that a specific alternative will lead to a favorable outcome, a profit, and that a positive expectation, or it would lead to negative consequences, a loss, and that is a negative expectation.

To conduct a PSI, the following steps must be followed^49^:

1. Identify the objective and determine the criteria and the alternatives.
2. Formulate a decision matrix with alternatives $\left({A}_{i}\right)$ as the rows, criteria $\left({C}_{i}\right)$ as the columns, and performance of alternatives versus criterion (attribute measures $\left({X}_{ij}\right)$) as the values.
3. Normalize the data in the matrix depending on the expectancy:
  - In the case of a positive expectancy, the normalization formula would be:
    15 $${R}_{ij}=\frac{{X}_{ij}}{{X}_{j}^{max}}$$
    b. Otherwise, in the case of negative expectancy, the formula would be:
    16 $${R}_{ij}=\frac{{X}_{j}^{min}}{{X}_{ij}}$$
    where Xij $\left(i=1, 2, \dots ,n and j=1, 2, \dots ,m\right)$ are the attribute measures in the decision matrix.
4. Calculate the Preference Variation value (PV_j_) using the following equation:
  17 $${\text{ P}V}_{j}={{\sum }_{i=1}^{N}[{R}_{ij}-{\overline{R} }_{J}]}^{2}$$
5. Calculate the deviation (Φ) in the preference value (PVj) for each attribute using the equation below:
  18 $$\Phi =1-{PV}_{j}$$
6. Calculate the overall preference value (Ψ) for each attribute using the equation below:
  19 $${\Psi }_{j}= \frac{{\Phi }_{j}}{{\sum }_{j=1}^{M}{\Phi }_{j}}$$
  Note that the sum of the preference value of all the attributes should be equal to 1.
7. Calculate the preference selection index (PSI) using the following equation:
  20 $${PSI}_{i}= {\sum }_{j=1}^{M}({R}_{ij }\times {\Psi }_{j})$$
8. Rank the alternatives based on their PSI values, where the best alternative is the one with the highest index.

### Sensitivity analysis

A sensitivity analysis was conducted on the four methods to avoid ambiguity and uncertainty while making a decision and to validate the consistency of the results. For the sake of simplicity, the sensitivity analysis will be applied to the two criteria with the highest weights for each MCDM method. The objective is to determine how changes in each criterion affect the final decision. We have chosen the extreme approach, where the weights should vary between − 50% and 50% of the nominal values. The results will be graphical, in the form of Tornado charts, to highlight the significance of the impact of the criteria on the final decision.

## Results and discussion

In this section, we will present the results of every MCDM method with a discussion of the ranked alternatives and the reasons behind such a rank. Since the first step of every method is to present the alternatives and the criteria, we will present them below to avoid redundancy. There are eight alternatives, and those are the cleaning methods mentioned previously: natural, manual, mechanical, robotic, coating, drone, electrical, and acoustic. On the other hand, there are seven criteria: capital cost, O&M cost, efficiency, cleaning time, water consumption, energy expenditure, and safety.

### AHP

**Figure 2:**
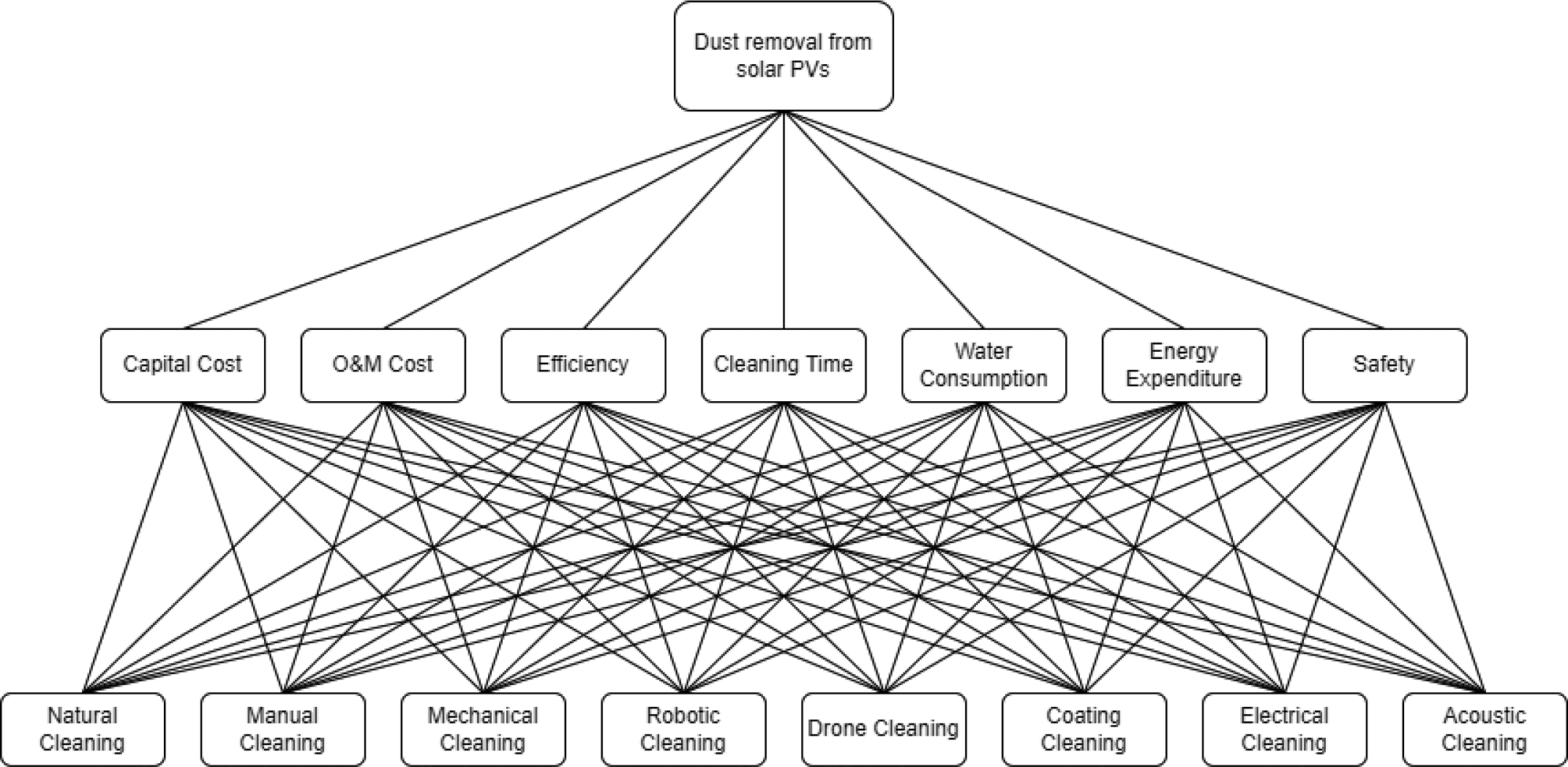
AHP hierarchy criteria and cleaning methods.

The first step of the AHP was to represent the goal, the criteria, and the alternatives as a hierarchy. Figure 2 illustrates the tree with the goal of dust cleaning as the roots, followed by the seven criteria as the upper level and the eight alternatives as the lower level. After that, a pairwise comparison between the criteria was carried out based on the survey results. Table 5 shows the pairwise matrix that shows how many times the criterion in each row is more important than the one in each column. For example, capital cost is 1.5 times more critical than O&M costs, twice more important than cleaning efficiency, three times more important than cleaning time, four times more important than water consumption, five times more important than energy expenditure, and six times more important than safety. It is noteworthy to mention that the pairwise matrix is diagonal, as can be seen.

**Table 5:** AHP Original Pairwise Matrix.

	Cap cost	O&M cost	Efficiency	Cleaning time	Water cons	Energy exp	Safety
Cap cost	1	1.5	2	3	4	5	6
O&M cost	0.667	1	1.5	2	3	4	5
Efficiency	0.5	0.667	1	2	3	4	5
Cleaning time	0.333	0.5	0.5	1	2	3	4
Water cons	0.25	0.333	0.333	0.5	1	2	3
Energy exp	0.2	0.25	0.25	0.333	0.5	1	3
Safety	0.167	0.2	0.2	0.25	0.333	0.333	1
**Total**	**3.12**	**4.45**	**5.78**	**9.08**	**13.83**	**19.33**	**27**

Step number 3 was to find the weight of every criterion. We first have to find the relative weight per column to do that. That is done by dividing every value on the matrix by the column’s total. After that, we calculate the row average, which are the criteria’s weights as shown in Table 6. Those values represent the weight of each selected criterion as a fraction of the total weight of 1.

**Table 6:** Pairwise matrix with weighted criteria.

	Cap cost	O&M cost	Efficiency	Cleaning time	Water cons	Energy exp	Safety	**Wi**
Cap cost	0.32	0.34	0.35	0.33	0.29	0.26	0.22	**0.30**
O&M cost	0.21	0.22	0.26	0.22	0.22	0.21	0.19	**0.22**
Efficiency	0.16	0.15	0.17	0.22	0.22	0.21	0.19	**0.19**
Cleaning time	0.11	0.11	0.09	0.11	0.14	0.16	0.15	**0.12**
Water cons	0.08	0.07	0.06	0.06	0.07	0.10	0.11	**0.08**
Energy exp	0.06	0.06	0.04	0.04	0.04	0.05	0.11	**0.06**
Safety	0.05	0.04	0.03	0.03	0.02	0.02	0.04	**0.03**

As seen from the table, the capital cost is the criterion with the highest weight and is, therefore, the most important one. This was initially highlighted in Table 5 following the survey results, as it was more important than all the other criteria. Additionally, the least essential criterion is safety, as the practice of dust cleaning is, in general, a very safe process regardless of the method.

The pairwise matrix is normalized later by multiplying the weights by the original pairwise matrix. We take the sum of each row and divide it by the corresponding weight of the criterion. The average of the resulting values is the largest eigenvalue ${\lambda }_{max}$. The values of ${\lambda }_{max}$, the consistency index and the consistency ratio are calculated below. According to the values in Table 3, the Random Index, RI, for a matrix of size n = 7 is 1.35. Since the CR is less than 0.1, then it is acceptable and the consistency level is reasonable, therefore we may continue with the decision-making process using AHP.

$${\lambda }_{max}=\frac{7.25+7.26+7.29+7.23+7.17+7.04+7.08}{7} = 7.188$$

$$C.I=\frac{{\lambda }_{max}-n}{n-1}= \frac{7.188-7}{7-1}=0.0313$$

$$C.R=\frac{C.I}{R.I}= \frac{0.0314}{1.35}=0.0232$$

After that, we proceed to conduct a pairwise comparison of the alternatives concerning each criterion based on the survey results. The comparison was on a scale from 1 to 9, where 1 is the least value such that both alternatives achieve the same level of cost or performance when compared to each other, and 9 is the highest value such that the first alternative is more important than the latter. For example, in our case, we consider that the capital cost of natural cleaning is 9 times less than all other alternatives, etc. After completing the pairwise comparison matrix for all the alternatives vs. each criterion, we find the normalized matrix and then calculate the weight of each alternative for each criterion. Following that, we sum product the criteria’s weights and the alternatives’ weights for each criterion; the product is the scores of the alternatives. Finally, we rank the alternatives based on their scores from largest to smallest. The weights of the alternatives versus the criteria and the alternatives scores and ranking are presented in Tables 7 and 8, respectively.

**Table 7:** Weights of each alternative for each criterion.

	Cap cost	O&M cost	Efficiency	Cleaning time	Water cons	Energy exp	Safety
Natural	0.48	0.48	0.02	0.29	0.33	0.26	0.48
Manual	0.17	0.04	0.37	0.12	0.08	0.26	0.02
Mechanical	0.10	0.06	0.04	0.29	0.02	0.04	0.13
Robotic	0.04	0.08	0.14	0.06	0.06	0.06	0.06
Drone	0.05	0.11	0.03	0.11	0.04	0.08	0.08
Coating	0.12	0.18	0.24	0.04	0.11	0.26	0.16
Electrical	0.02	0.02	0.09	0.02	0.33	0.02	0.03
Acoustic	0.03	0.03	0.07	0.15	0.02	0.03	0.03

**Table 8:** Scores and ranking of the alternatives.

Alternatives	Score	Overall Rank
Natural	0.35	1
Manual	0.17	2
Coating	0.15	3
Mechanical	0.09	4
Robotic	0.07	5
Drone	0.07	6
Electrical	0.06	7
Acoustic	0.05	8

### QFD

**Figure 3:**
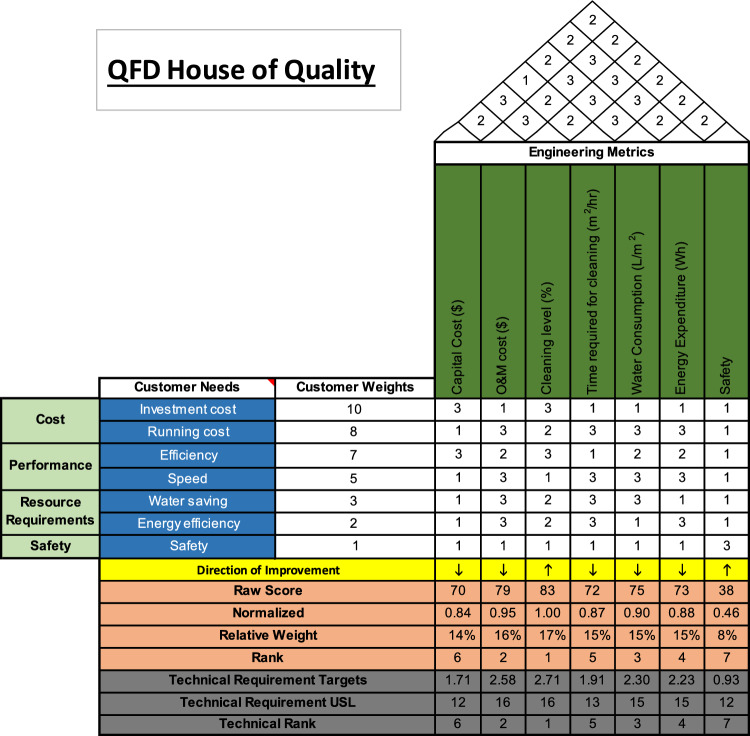
QFD House of Quality customer needs vs engineering metrics.

Before performing the QFD analysis, we define the customer needs and engineering metrics. Customer needs are the criteria in the customer’s language and are divided into four main categories: cost, which includes investment costs and running costs; performance, which includes efficiency and speed; resource requirements, including water saving and energy efficiency; and safety. On the other hand, the engineering metrics are the seven criteria mentioned previously. After that, we built the QFD House of Quality (HOQ), shown in Fig. 3 below. The customer weights are assigned by the customer in order of importance from 1 to 10, where 1 is the least important and 10 is the most important. As can be seen, safety is the least important requirement, as was the case before in AHP, and the capital cost is the most important one. Thereafter, the relationship between the customer needs and the engineering requirements is assigned a value of 1, 2, or 3, where 1 represents a weak relationship, 2 is a medium relationship, and three is a strong one. Moreover, the interrelationships between the engineering metrics are mapped on the roof of the HOQ between 1 and 3, where 1 indicates a negative relationship between the two metrics, three a positive relationship between the two metrics, and when the relationship is not specific, we assign a value of 2.

After defining the weights of the customer needs, their relationships with the engineering metrics, and the interrelationships between the engineering metrics, we proceed to calculate the targets to rank the criteria. The direction of improvement illustrates whether we would like the metric to be high or low. For example, it would be better if the costs and water consumption were lower. Similarly, a higher level of efficiency and cleaning is desired. First, we find the raw scores; then, we normalize them by dividing each raw score by the maximum raw score (in this case, 83). After that, we calculate the relative weights and then rank the engineering requirements (i.e., criteria) from highest weight to lowest. As can be seen, the cleaning level ranked first, while the safety level was ranked last.

To illustrate the impact of the interrelationships between the engineering requirements, we calculate the Technical Requirements Upper Specification Limit (USL) as the sum of the values related to each metric. Multiplying that value by the relative weight gives the Technical Requirement Targets, based on which we rank the criteria again. As can be seen, the ranking is consistent.

**Figure 4:**
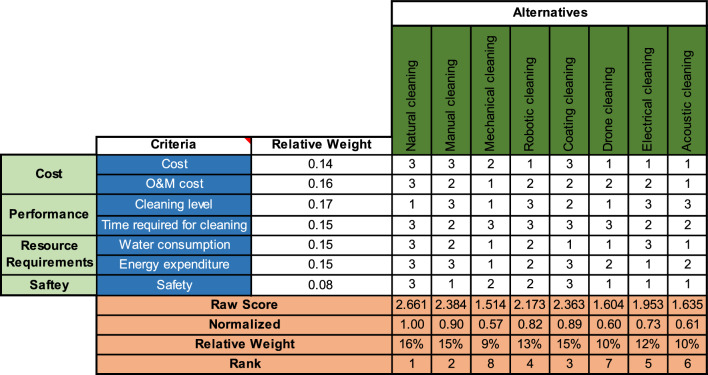
HOQ criteria against alternatives.

After that, the criteria are mapped against the alternatives in another HOQ, as can be seen in Fig. 4, using the relative weights from the previous figure. Similar to before, we define the relationships between the alternatives and the criteria and assign values between 1 and 3 to quantify those relationships. In this case, the relationship indicates the performance of each alternative versus each criterion, where 1 represents a poor performance and three an excellent one. Similarly, we compute the raw and normalized scores and the relative weights and conclude the ranking of the alternatives. Using QFD, natural cleaning is the best alternative, followed by manual and surface coating, whereas mechanical cleaning is the least optimal.

### FTOPSIS

Unlike AHP, FTOPSIS relies on triplets of fuzzy real numbers to translate human judgment rather than crisp numbers^46^. More importantly, this method is easier to translate as it allows the use of linguistic terms from decision-makers instead of weights or degrees of importance like other MCDM methods. Just as was presented in Table 4 in the methodology, the raw information gathered from the survey in linguistic terms is transformed into fuzzy numbers.

Tables 9 and 10 present the experts’ judgments on solar and sustainable energy through the surveys and their equivalents in fuzzy numbers, respectively. It is noteworthy to mention that row ‘weightage’ refers to the weights assigned to the criteria on top. In addition, Table AF presents the minimum and maximum values of columns 1 and 3, ${a}_{i}$ and ${c}_{j}$ respectively, depending on whether the criterion is a benefit or cost one.

**Table 9:** Linguistic Decision Matrix.

	Cap cost	O&M cost	Efficiency	Cleaning time	Water cons	Energy exp	Safety
**Weightage**	**Very high**	**Very high**	**High**	**Medium**	**Low**	**Low**	**Very low**
Natural	Very high	Very High	Very low	Very high	Very high	Very high	Very high
Manual	High	High	Very high	Medium	Medium	Very high	Very low
Mechanical	Medium	Low	Low	Very high	Low	Low	High
Robotic	Low	Medium	High	Medium	Medium	Medium	Medium
Drone	Low	Medium	Low	High	Low	Medium	Medium
Coating	High	Very high	Very high	Low	Medium	Very high	Very high
Electrical	Very low	Low	Medium	Very low	Very high	Very low	Low
Acoustic	Very low	Low	Medium	Very low	Very low	Low	Low

**Table 10:** Fuzzy decision matrix.

	Cap cost	O&M cost	Efficiency	Cleaning time	Water cons	Energy exp	Safety
**Weightage**	**7,9,9**	**7,9,9**	**5,7,9**	**3,5,7**	**1,3,5**	**1,3,5**	**1,1,3**
Natural	7,9,9	7,9,9	1,1,3	7,9,9	7,9,9	7,9,9	7,9,9
Manual	5,7,9	5,7,9	7,9,9	3,5,7	3,5,7	7,9,9	1,1,3
Mechanical	3,5,7	1,3,5	1,3,5	7,9,9	1,3,5	1,3,5	5,7,9
Robotic	1,3,5	3,5,7	5,7,9	3,5,7	3,5,7	3,5,7	3,5,7
Drone	1,3,5	3,5,7	1,3,5	5,7,9	1,3,5	3,5,7	3,5,7
Coating	5,7,9	7,9,9	5,7,9	1,3,5	3,5,7	7,9,9	7,9,9
Electrical	1,1,3	1,3,5	3,5,7	1,1,3	7,9,9	1,1,3	1,3,5
Acoustic	1,1,3	1,3,5	3,5,7	1,1,3	1,1,3	1,3,5	1,3,5
${{\varvec{a}}}_{{\varvec{i}}}$	**1**	**1**		**1**	**1**	**1**	
${{\varvec{c}}}_{{\varvec{j}}}$			**9**				**9**

After that, we calculate the normalized fuzzy decision matrix by dividing ${a}_{i}$ by the fuzzy numbers of the cost criteria and dividing the fuzzy numbers of the benefit criteria by ${c}_{j}$. The resulting matrix is presented in Table 11. We multiply the normalized fuzzy matrix by the weights of the criteria to get the weighted normalized fuzzy decision matrix that is presented in Table 12. In addition, we define the FPIS ${(A}^{*})$ and FNIS ${(A}^{-})$, as the maximum and minimum fuzzy values per column, respectively.

**Table 11:** Normalized fuzzy decision matrix.

	Cap cost	O&M cost	Efficiency	Cleaning time	Water cons	Energy exp	Safety
Natural	0.14,0.11,0.11	0.14,0.11,0.11	0.11,0.11,0.33	0.14,0.11,0.11	0.14,0.11,0.11	0.14,0.11,0.11	0.78,1,1
Manual	0.2,0.14,0.11	0.2,0.14,0.11	0.78,1,1	0.33,0.2,0.14	0.33,0.2,0.14	0.14,0.11,0.11	0.11,0.11,0.33
Mechanical	0.33,0.2,0.14	1,0.33,0.2	0.11,0.33,0.56	0.14,0.11,0.11	1,0.33,0.2	1,0.33,0.2	0.56,0.78,1
Robotic	1,0.33,0.2	0.33,0.2,0.14	0.56,0.78,1	0.33,0.2,0.14	0.33,0.2,0.14	0.33,0.2,0.14	0.33,0.56,0.78
Drone	1,0.33,0.2	0.33,0.2,0.14	0.11,0.33,0.56	0.2,0.14,0.11	1,0.33,0.2	0.33,0.2,0.14	0.33,0.56,0.78
Coating	0.2,0.14,0.11	0.14,0.11,0.11	0.56,0.78,1	1,0.33,0.2	0.33,0.2,0.14	0.14,0.11,0.11	0.78,1,1
Electrical	1,1,0.33	1,0.33,0.2	0.33,0.56,0.78	1,1,0.33	0.14,0.11,0.11	1,1,0.11	0.11,0.33,0.56
Acoustic	1,1,0.33	1,0.33,0.2	0.33,0.56,0.78	1,1,0.33	1,1,0.33	1,0.33,0.2	0.11,0.33,0.56

**Table 12:** Weighted normalized fuzzy decision matrix.

	Cap cost	O&M cost	Efficiency	Cleaning time	Water cons	Energy exp	Safety
Natural	1,1,1	1,1,1	0.56,0.78,3	0.43,0.56,0.78	0.14,0.33,0.56	0.14,0.33,0.56	0.78,1,3
Manual	1.4,1.29,1	1.4,1.29,1	3.89,7,9	1,1,1	0.33,0.6,0.71	0.14,0.33,0.56	0.11,0.11,1
Mechanical	2.33,1.8,1.29	7,3,1.8	0.56,2.33,5	0.43,0.56,0.78	1,1,1	1,1,1	0.56,0.78,3
Robotic	7,3,1.8	2.33,1.8,1.29	2.78,5.44,5	1,1,1	0.33,0.6,0.71	0.33,0.6,0.71	0.33,0.56,2.33
Drone	7,3,1.8	2.33,1.8,1.29	0.56,2.33,5	0.6,0.71,0.78	1,1,1	0.33,0.6,0.71	0.33,0.56,2.33
Coating	1.4,1.29,1	1,1,1	2.78,5.44,9	3,1.67,1.4	0.33,0.6,0.71	0.14,0.33,0.56	0.78,1,3
Electrical	7,9,3	7,3,1.8	1.67,3.89,7	3,5,2.33	0.14,0.33,0.56	1,3,1.67	0.11,0.33,1.67
Acoustic	7,9,3	7,3,1.8	1.67,3.89,7	3,5,2.33	1,3,1.67	1,1,1	0.11,0.33,1.67
A*	**7,9,3**	**7,3,1.8**	**3.89,7,9**	**3,5,2.33**	**1,3,1.67**	**1,3,1.67**	**0.78,1,3**
**A-**	**1,1,1**	**1,1,1**	**0.56,0.78,3**	**0.43,0.56,0.78**	**0.14,0.33,0.56**	**0.14,0.33,0.56**	**0.11,0.11,1**

Following that, we calculate the distance of each alternative to the positive and negative ideal solution using the Euclidean distance. The distances from each alternative to the positive ideal solution are presented in Table 13, and those to the negative ideal solution in Table 14.

**Table 13:** Fuzzy positive ideal solution matrix.

	Cap cost	O&M cost	Efficiency	Cleaning time	Water cons	Energy exp	Safety	di*
Natural	5.8878	3.6806	5.3487	3.0976	1.7398	1.7398	0.0000	21.4943
Manual	5.6235	3.4127	0.0000	2.6943	1.5396	1.7398	1.3209	16.3308
Mechanical	5.0516	0.0000	4.0369	3.0976	1.2172	1.2172	0.1814	14.8018
Robotic	3.5327	2.7978	1.1037	2.6943	1.5396	1.5396	0.5290	13.7367
Drone	3.5327	2.7978	4.0369	2.9747	1.2172	1.5396	0.5290	16.6279
Coating	5.6235	3.6806	1.1037	1.9985	1.5396	1.7398	0.0000	15.6857
Electrical	0.0000	0.0000	2.4911	0.0000	1.7398	0.0000	0.9428	5.1737
Acoustic	0.0000	0.0000	2.4911	0.0000	0.0000	1.2172	0.9428	4.6511

**Table 14:** Fuzzy negative ideal solution matrix.

	Cap cost	O&M cost	Efficiency	Cleaning time	Water cons	Energy exp	Safety	**di-**
Natural	0.0000	0.0000	0.0000	0.0000	0.0000	0.0000	1.3209	1.3209
Manual	0.2838	0.2838	5.3487	0.4372	0.2102	0.0000	0.0000	6.5638
Mechanical	0.9128	3.6806	1.4628	0.0000	0.6774	0.6774	1.2439	8.6549
Robotic	3.6806	0.9128	4.5722	0.4372	0.2102	0.2102	0.8215	10.8448
Drone	3.6806	0.9128	1.4628	0.1349	0.6774	0.2102	0.8215	7.9002
Coating	0.2838	0.0000	4.5722	1.6567	0.2102	0.0000	1.3209	8.0439
Electrical	5.8878	3.6806	2.9952	3.0976	0.0000	1.7398	0.4057	17.8067
Acoustic	5.8878	3.6806	2.9952	3.0976	1.7398	0.6774	0.4057	18.4841

Finally, we compute the closeness coefficient $({CC}_{i})$ and rank the alternatives in descending order. The alternative with the highest relative closeness value is selected as the best option. Table 15 summarizes the calculated ${CC}_{i}$ for each cleaning method. Unlike the two previous models, the best alternative is the acoustic alternative in the case, whereas natural cleaning is the least favorite.

**Table 15:** Closeness coefficients and ranking of alternatives.

	di*	di-	Cci	Rank
Natural	21.4943	1.3209	0.0579	8
Manual	16.3308	6.5638	0.2867	7
Mechanical	14.8018	8.6549	0.3690	4
Robotic	13.7367	10.8448	0.4412	3
Drone	16.6279	7.9002	0.3221	4
Coating	15.6857	8.0439	0.3390	3
Electrical	5.1737	17.8067	0.7749	2
Acoustic	4.6511	18.4841	0.7990	1

### PSI

After defining the criteria and the alternatives, a decision matrix is created based on the average score point provided by the experts in the field of sustainability and solar energy via the surveys. We categorize the criteria based on their expectancy, similar to the direction of improvement in the QFD analysis, where if a criterion reflects a better performance (positive expectancy), then we assign it a grade ‘B’ for beneficial, whereas ‘NB’ represents non-beneficial for poor performance criteria (negative expectancy). The decision matrix with the scores and the attributes is highlighted in Table 16 below. The minimum or maximum rating depends on whether the criterion is beneficial or non-beneficial.

**Table 16:** Decision Matrix of the alternatives versus criteria.

	Cap cost	O&M cost	Efficiency	Cleaning time	Water cons	Energy exp	Safety
Attribute	NB	NB	B	NB	NB	NB	B
Natural	1	1	1	3	1	1	9
Manual	2	2	9	5	4	2	2
Mechanical	6	5	3	1	9	6	4
Robotic	6	4	6	5	5	5	4
Drone	5	3	4	5	6	4	5
Coating	3	4	7	3	3	1	7
Electrical	8	7	5	6	1	9	4
Acoustic	7	6	5	6	6	7	4
**Max/Min**	**1**	**1**	**9**	**1**	**1**	**1**	**9**

After that, we normalize the data in the matrix depending on the attribute, whether it’s B or NB, and we calculate the mean normalized value of each criterion, ${\overline{R} }_{J}$, which is the average column value. The resulting matrix is shown in Table 17.

**Table 17:** Normalized Decision Matrix.

	Cap cost	O&M cost	Efficiency	Cleaning time	Water cons	Energy exp	Safety
Attribute	NB	NB	B	NB	NB	NB	B
Natural	1.000	1.000	0.111	0.333	1.000	1.000	1.000
Manual	0.500	0.500	1.000	0.200	0.250	0.500	0.222
Mechanical	0.167	0.200	0.333	1.000	0.111	0.167	0.444
Robotic	0.167	0.250	0.667	0.200	0.200	0.200	0.444
Drone	0.200	0.333	0.444	0.200	0.167	0.250	0.556
Coating	0.333	0.250	0.778	0.333	0.333	1.000	0.778
Electrical	0.125	0.143	0.556	0.167	1.000	0.111	0.444
Acoustic	0.143	0.167	0.556	0.167	0.167	0.143	0.444
${\overline{{\varvec{R}}} }_{{\varvec{J}}}$	**0.329**	**0.355**	**0.556**	**0.325**	**0.403**	**0.421**	**0.542**

As steps 4, 5, and 6 in the methodology dictate, we calculate the Preference Variation value (PV_j_), the deviation (Φ), and the overall preference value (Ψ) for each attribute. The values are summarized in Table 18.

**Table 18:** Summary of *PVj*, Φ, and Ψ values.

	Cap cost	O&M cost	Efficiency	Cleaning time	Water cons	Energy exp	Safety
PVj	0.625	0.564	0.519	0.553	0.979	0.993	0.406
*Φ*	0.375	0.436	0.481	0.447	0.021	0.007	0.594
Ψ	0.159	0.185	0.204	0.189	0.009	0.003	0.252

Following that, we find the Preference Selection Index ${(PSI}_{i})$ by multiplying the normalized data in the matrix in Table 19 by the overall preference value (Ψ). The total of each row is the PSI per alternative. Finally, we rank the alternatives based on their PSI scores, and as can be seen, the optimal method is natural cleaning, followed by surface coating, followed by manual cleaning. In contrast, acoustic cleaning is the least optimal cleaning method.

**Table 19:** Summary of the PSI and ranking of the alternatives.

	Cap cost	O&M cost	Efficiency	Cleaning time	Water cons	Energy exp	Safety	**PSI**_**i**_	**Rank**
Natural	0.159	0.185	0.023	0.063	0.009	0.003	0.252	**0.693**	**1**
Manual	0.079	0.092	0.204	0.038	0.002	0.002	0.056	**0.473**	**3**
Mechanical	0.026	0.037	0.068	0.189	0.001	0.001	0.112	**0.434**	**4**
Robotic	0.026	0.046	0.136	0.038	0.002	0.001	0.112	**0.361**	**6**
Drone	0.032	0.062	0.091	0.038	0.001	0.001	0.140	**0.364**	**5**
Coating	0.053	0.046	0.159	0.063	0.003	0.003	0.196	**0.522**	**2**
Electrical	0.020	0.026	0.113	0.032	0.009	0.000	0.112	**0.312**	**7**
Acoustic	0.023	0.031	0.113	0.032	0.001	0.000	0.112	**0.312**	**8**

### Sensitivity analysis

#### AHP

**Figure 5:**
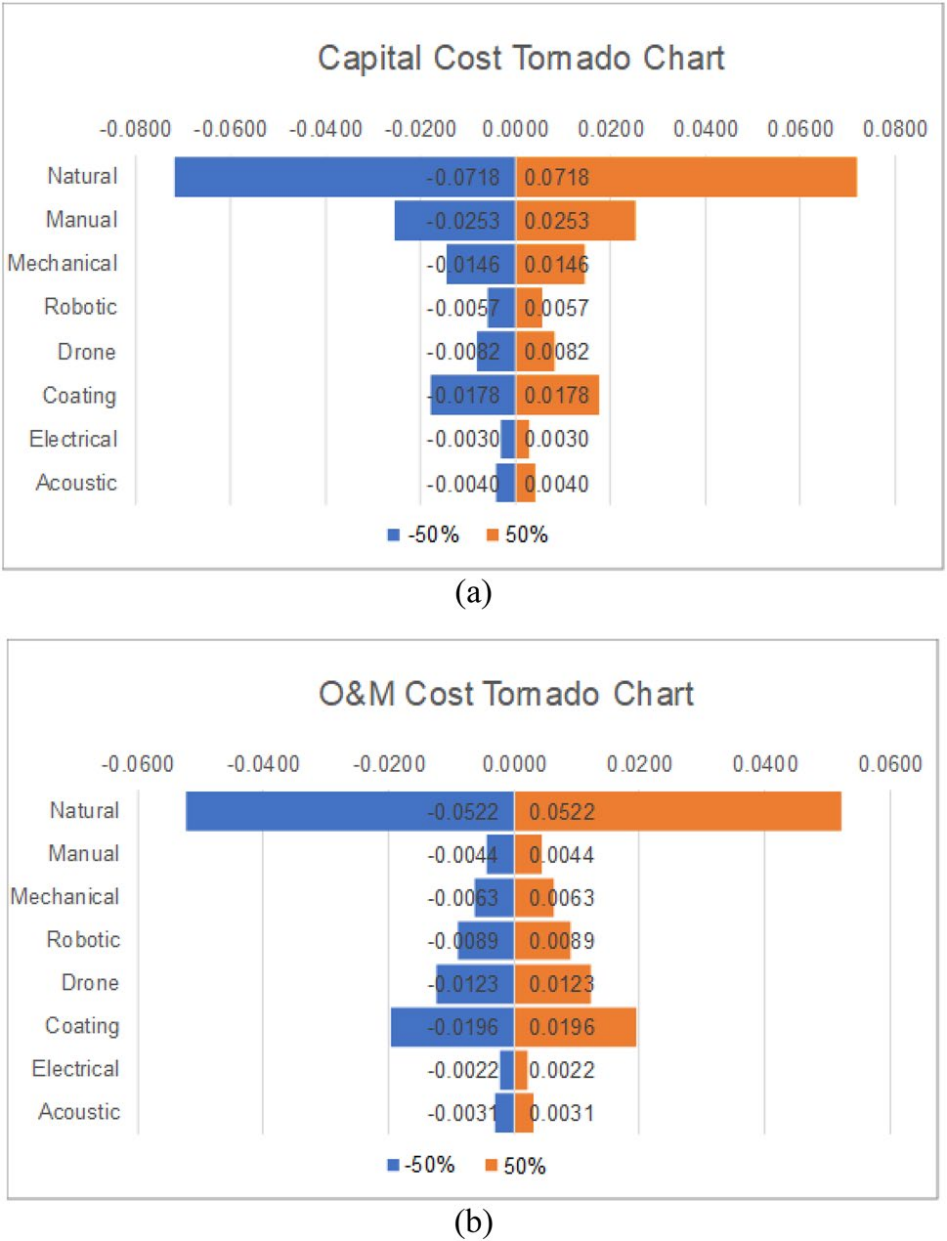
Tornado charts for AHP: (**a**) Capital cost and (**b**) O&M.

The weights of the criteria shown in Table 6 indicate that capital cost is the most influential criterion for the AHP method. As such, it weighs 30% of the final decision, therefore, any uncertainty related to its weight plays a major role in the final decision. When −/ + 50% uncertainty was applied to it, the AHP scores changed, but the ranking barely changed as the top three methods remained the same, with the natural cleaning method first, then manual cleaning, and then surface coating. Figure 5a shows the impact of a −/ + 50% change on the capital cost alone, all other weights kept constant, on the different cleaning techniques. As can be seen, the natural method is the most impacted one because its weight compared to the capital cost is 0.48, as shown in Table 7, and that was the highest weight compared to other alternatives compared to capital cost. An upward or downward 50% change in the weight of the capital cost impacts the natural cleaning decision by an upward or downward 20%, respectively. That is, any change in the capital weight will majorly impact decisions related to natural cleaning, manual cleaning, and then surface coating. Regarding the small weights of other alternatives compared to the capital cost, it is normal to notice that the impact is minimal.

The second most influential criterion is the cost of O&M with 0.22. Figure 5b shows the impact of a −/ + 50% change on the O&M cost alone, all other weights kept constant, on the different cleaning techniques. Similar to the capital cost, the weight of the natural cleaning for O&M cost is 0.48, which also makes it the highest rate of that column. This justifies the long band next to Natural in the figure. Regarding the lower weight of O&M costs, the −/ + 50% changes the score of natural cleaning by −/ + 15% only. Changes in O&M costs impact highly surface coating and drone cleaning as well as can be seen in that chart.

#### QFD

**Figure 6:**
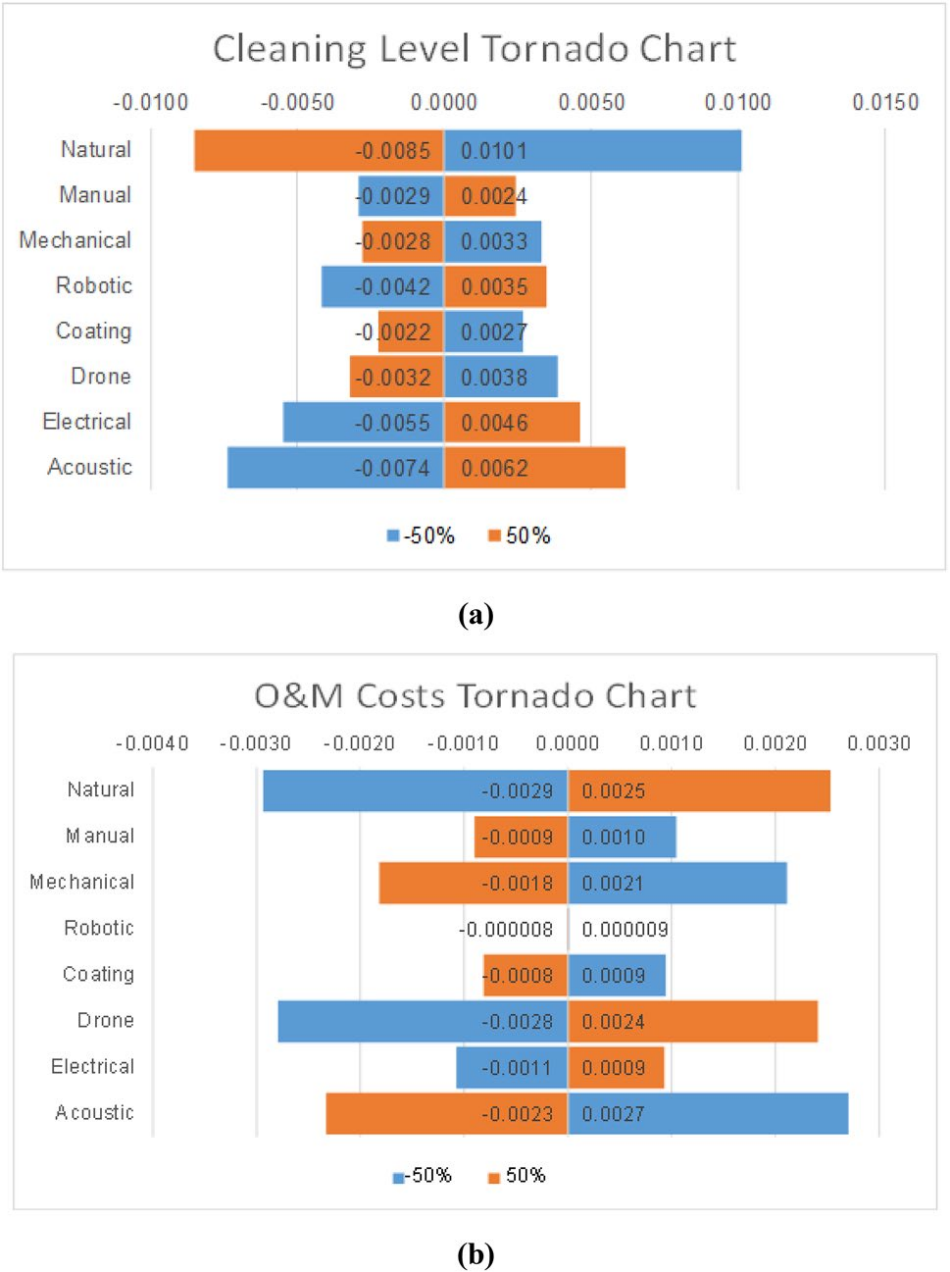
Tornado charts for QFD: (**a**) Cleaning Level and (**b**) O&M.

The sensitivity analysis in the QFD method focused on the relative weights of the criteria from the House of Quality in Fig. 3. The ranking of the weights shows that the cleaning level, followed by the O&M cost, are the most important criteria when selecting an optimal cleaning method for the solar panels. Consequently, the sensitivity analysis will be focused on these two criteria. Figure 6a illustrates the impact of increasing and decreasing the weight of the cleaning level criterion by 50%. On the other hand, Fig. 6b highlights the change in the scores of the alternatives following a change in the O&M cost by −/ + 50%. Similar to the AHP method, the changes in both criteria impact the natural cleaning method more than the rest. That could be linked to the fact that natural cleaning ranks first in the HOQ of the criteria against the alternatives in Fig. 4. However, it is noticeable that the differences are very small between the sensitivity analysis values and the nominal values, which explains the same ranking of the alternatives. While the change in the cleaning level seems to have a steady impact on the cleaning methods, the change in O&M costs seems more vibrant on a smaller scale. In other words, an increase or decrease in the O&M costs impacts highly most of the cleaning methods, except for the robotic method where there was almost no change. That could be due to the fact that O&M costs are not an important factor when studying robotic cleaning. It might be that other factors influence that alternative.

More importantly, unlike the AHP sensitivity analysis, increases in the weights of the selected factors did not necessarily increase the scores of the cleaning methods. As such, an increase in the cleaning level results in a lower score for natural cleaning, mechanical cleaning, surface coating, and drone cleaning, and vice-versa. The same logic applies to changes in the O&M costs. This means that changes in those criteria are inversely related to the just mentioned cleaning methods. This can be due to the fact that maybe the threshold for these methods was exceeded, or that at that level of change, the cleaning level becomes ineffective and the O&M costs become non-cost-efficient.

#### FTOPSIS

**Figure 7:**
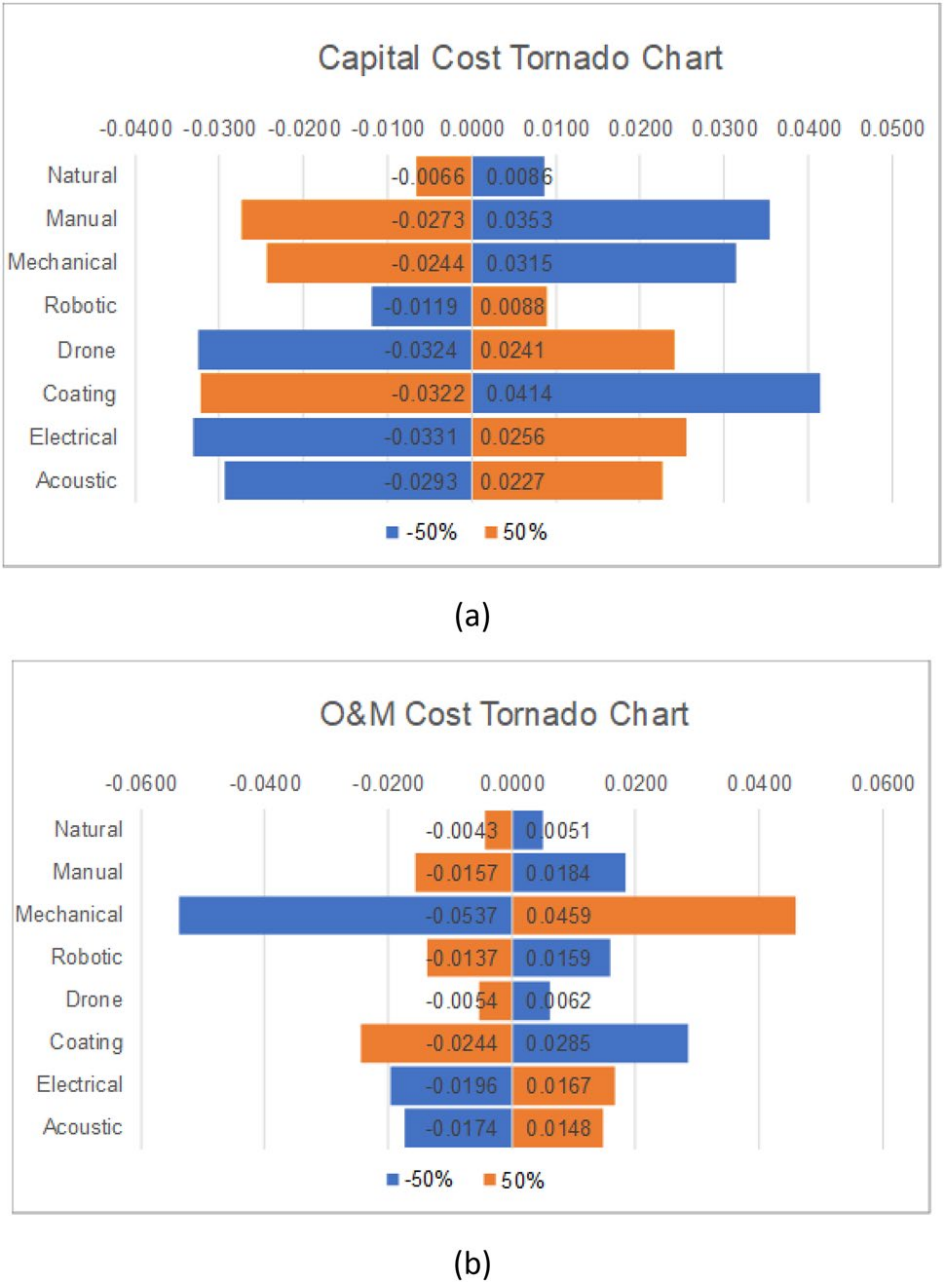
Tornado charts for FTOPSIS: (**a**) Capital cost and (**b**) O&M.

Performing sensitivity analysis for the FTOPSIS method was a little different, considering the weights are in the form of fuzzy numbers. Therefore, the changes were applied to the triplet as a whole. That is, the three numbers were changed simultaneously. The two criteria that have the highest rating are the capital cost and the O&M cost; thus, they were both considered for the analysis. Again, the ranking of the alternatives remained intact despite the 50% decrease/increase in both criteria. Figure 7a illustrates the changes in the fuzzy weight of the capital cost on the final ${CC}_{i}$, whereas Fig. 7b shows the effect of changing the O&M cost’s fuzzy weights on the ${CC}_{i}$ of the cleaning techniques. As can be seen, the changes in the capital cost have a bigger impact on electrical, drone, coating, acoustic, and manual cleaning methods. In contrast, the change in the O&M cost heavily impacts mechanical cleaning. Not only that, but the impact of these changes on the capital cost is inversely proportional to the closeness coefficient of natural, manual, mechanical, and surface coating. As mentioned before, this could be due to overpassing the capital cost threshold, especially since those methods are mature in the market and their capital costs are not as high as the other methods. The same thing applies to mechanical cleaning with changes in O&M costs. As can be seen, the difference is large compared to the other methods, and that could be because the O&M costs for mechanical cleaning are low and not very important when deciding on solar panel cleaning methods.

#### PSI

**Figure 8:**
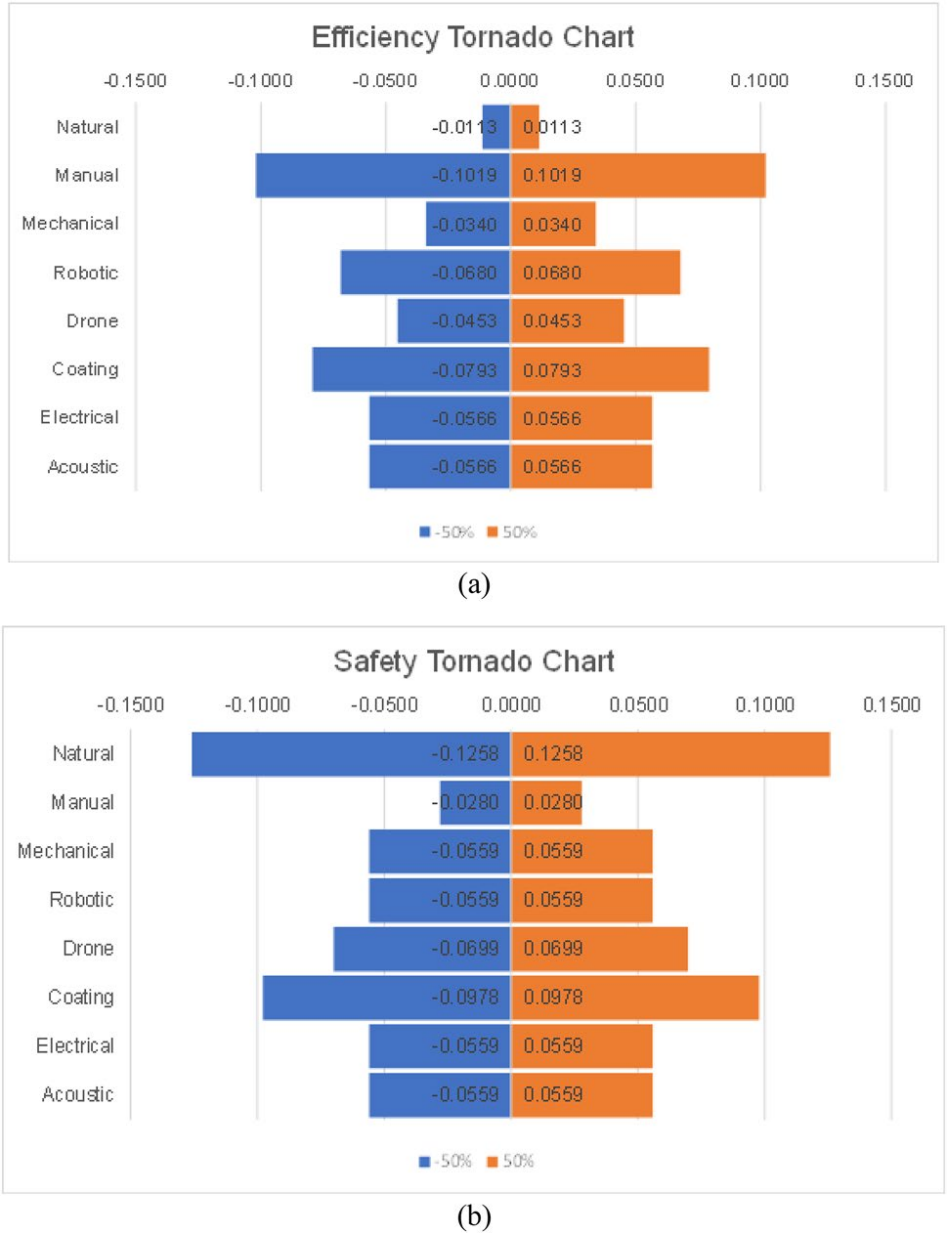
Tornado charts for PSI: (**a**) Efficiency and (**b**) Safety.

The sensitivity analysis for the PSI method was performed by changing the weights of the two most important criteria in the analysis. Based on the ${\Psi }_{j}$ values in Table 18, efficiency and safety, are the most important criteria when deciding which cleaning method to opt for. Figure 8a illustrates the impact of increasing and decreasing the efficiency weight by 50%, and Fig. 8b shows the sensitivity of the various cleaning methods when a −/ + 50% change occurs in the safety weight. Both figures show a steady change throughout the methods. That can be because the criteria weights are more or less uniform, between 0.15 and 0.25, except for water consumption and energy expenditure. This means that the weights of safety (0.25) and efficiency (0.20) should have a minimal impact on the alternatives. Indeed, since manual cleaning seems to be the most influenced by changes in efficiency, a 50% decrease in that only decreases the alternative’s PSI by 21%. Similarly, a 50% increase in the safety weight increases the PSI of the natural cleaning alternative by 18% only. This means that although these two criteria have the highest weights, their impact is still minor on the choice of the optimal alternative. To confirm that, the ranking of the alternatives remained steady for both sensitivity analyses. Here as well, it can be seen that the scale of change is small since the difference between the sensitivity values and the nominal values is small throughout.

## Discussion

While the AHP, QFD, and PSI agree that natural cleaning, manual cleaning, and surface coating are the best methods for dust cleaning from solar PVs in the hot, humid climate zone (e.g., most coastal cities in the Arab Gulf region), FTOPSIS has another say. Using this latter, natural cleaning was the last of the ranking as it was the farthest from the positive ideal solution. That could be due to the normalization of the decision matrix, as the FTOPSIS method takes into account the direction of improvement and considers fuzzy sets that reflect uncertainty. In addition, that difference could be due to the weighting of criteria as the FTOPSIS uses fuzzy weights instead of explicit crisp values. As for the three other methods, AHP, QFD, and PSI, the top three cleaning methods were the same: natural cleaning, manual cleaning, and surface coating. The natural cleaning method outperformed all the other cleaning methods, and that is reasonable as it involves less costs and resources and a higher safety level, as well as its capacity to clean large areas such as solar farms and centralized solar systems^1,2^. For manual cleaning, the capital and running costs of this method are low to moderate, but it is considered the best in terms of cleaning level compared to other techniques^1,4^. Surface coatings are considered a very good option due to reasonable capital and maintenance costs since coatings can last for a very long time before being replaced. Also, its major advantage is that it reduces the time required between cleanings due to its ability to reduce the amount of dust accumulated on the panel, and that increases solar irradiance^17,38,40^. It is noteworthy to mention that the cleaning methods analyzed do not provide any idea about the loss of power from PV. As such, each method is considered individually regardless of its impact on the generated power output.

Although acoustic cleaning is still at the research stage, the FTOPSIS seems to find it the most favorable cleaning method. As positive as that sounds, that result does not really reflect a major benefit of using acoustic cleaning because there is still very limited information about its applicability, efficacy, and consistency in the literature. Moreover, most people are still not familiar with it, neither with drone or electrical cleaning methods, including the experts who filled out our surveys. In addition, the economic feasibility of it is questionable since the estimated capital cost and O&M costs are very high compared to the other methods. Finally, there is still some uncertainty about the scale to which the cleaning systems are implementable. Nonetheless, these novel and innovative cleaning methods are being extensively studied and could be commercialized soon, considering a major drop in their capital costs. Our results are in line with what exists in the literature in the sense that manual cleaning is among the best alternatives. As discussed in the introduction, Obeidat^12^ and Aljaghoub et al.^41^ both concluded that manual cleaning was the best option in Jordan and UAE, respectively. Also in the UAE, AlMallahi et al.^40^ showed using TOPSIS that manual cleaning is the second best alternative after robotic cleaning. This comparison validates our results in a way since they did not include all the cleaning methods, namely natural cleaning. Table 20 summarizes the results of the different MCDM methods used, along with the strengths and weaknesses of each method. It is important to note that different methods may yield varying results. This was evident with FTOPSIS, which produced slightly different outcomes compared to the other methods. However, this is not a drawback of the model, rather, it demonstrates that the use of multiple MCDM methods can aid decision-makers in gaining a deeper understanding of the tradeoffs, leading to a data-driven selection decision for solar farms.

**Table 20:** Summary of MCDM methods: features & results.

MCDM method	Features		Results
	Strengths	Weaknesses	
AHP	• Allows for the decomposition of complex decision problems into a hierarchy of criteria and alternatives • Provides a systematic and structured approach to decision-making	• Relies heavily on the subjective judgments of decision-makers in pairwise comparisons • Can be time-consuming and complex, especially when dealing with large decision problems	**1**^**st**^ rank: Natural cleaning **2**^**nd**^ rank: Manual cleaning **3**^**rd**^ rank: Surface coating
QFD	• Helps ensure that the technical requirements of a product or service align with the needs and wants of the customer • Can improve customer satisfaction by prioritizing customer needs and wants	• Requires extensive customer research and input, which can be time-consuming and expensive • Assumes that customer needs and wants are fixed and do not change over time	**1**^**st**^ rank: Natural cleaning **2**^**nd**^ rank: Manual cleaning **3**^**rd**^ rank: Surface coating
FTOPSIS	• Can handle imprecise and uncertain data through the use of fuzzy logic • Provides a flexible and customizable approach to decision-making • Provides a straightforward and easy-to-understand output	• Can be limited by the availability and quality of data • May not be suitable for decision problems that require precise or exact solutions	**1**^**st**^ rank: Acoustic cleaning **2**^**nd**^ rank: Electrical cleaning **3**^**rd**^ rank: Surface coating
PSI	• Quantifies subjective preferences objectively • Enhances transparency and objectivity in decision-making	• Calculation complexity can increase with larger datasets • Relies on accurate and reliable preference elicitation	**1**^**st**^ rank: Natural cleaning **2**^**nd**^ rank: Surface coating **3**^**rd**^ rank: Manual cleaning

The sensitivity analysis performed on the selected MCDM methods to check the consistency of our decisions serves its purpose. As such, the ranking of the alternatives, or at least the top 3 alternatives, remained the same for the four methods. The sensitivity analysis showed how heavy or minimal the impact of the most important criteria is on all the alternatives. The capital cost and the O&M costs were the most studied criteria regarding their importance and, therefore, high weights. The results show that change could be symmetric, as is the case for AHP and PSI, as well as asymmetric, such as FTOPSIS and QFD. That is because the analysis is linear for AHP and PSI, whereas it involves interrelationships between the criteria for QFD as well as uncertainty for FTOPSIS. In addition, it was shown that some discrepancies in the results could be because a threshold was surpassed, and therefore, we have reached inefficiency or that other criteria might influence certain alternatives differently. Overall, it can now be said with certainty that natural cleaning, manual cleaning, and surface coating are the best methods to clean dust off solar panels in Abu Dhabi or regions with similar climate conditions. Such findings concur with similar findings in other studies, for example^3,4,7,8,15,17^.

## Limitations

Of the limitations of this research is the small number of experts asked. That number might be small when using the QFD method; however, the results were logical and totally in line with other methods. In this research, we have decided to rely on quality rather than quantity, which is why only experts in the field were asked since personal judgment is crucial in this case. As was seen, all the results were based on the data collected from the surveys handed to the expert, while the rest was from the literature. This can limit the breadth and depth of the study’s conclusions, and therefore, caution should be exercised when interpreting the results.

Moreover, the study was conducted in a Middle Eastern country that is characterized by dry weather and limited rainfall. This does not necessarily imply that the results could be generalized to regions with the same weather since the conditions are not necessarily the same; for example, some dry regions do not experience heavy dust storms, as does Abu Dhabi. Also, the effectiveness of natural cleaning methods heavily relies on the availability and frequency of rainfall events. In regions with scant rainfall, natural cleaning may not be the most suitable option, potentially leading to subpar cleaning efficiency and compromised PV panel performance.

The criteria used in this study, including capital cost, operational cost, cleaning efficiency, cleaning time, water consumption, energy expenditure, and safety, were not specifically tailored to address the challenges faced in dry regions. One notable limitation was the inability to adequately adjust the cost criterion for natural cleaning, given that rainfall is universally available and does not incur a direct cost. Additionally, cleaning time can vary significantly in regions with limited rainfall, potentially impacting the ranking of natural cleaning methods.

To overcome those limitations and enhance the reliability of results, it is recommended that future studies incorporate region-specific criteria that focus on the characteristics of the region, namely rainfall. That could result in more accurate and applicable rankings for PV panel cleaning methods. Moreover, that could enable the identification and prioritization of alternative cleaning methods that are better suited to these specific environments. Accordingly, the number of alternatives to choose from would be lower, and the results would be more reliable and accurate.

## Conclusion and future work

Solar power holds great promise in reducing reliance on fossil fuels, and PV panels serve as a popular means of harnessing it. However, the accumulation of dust on these panels can significantly impede their efficiency, necessitating regular cleaning, which comes at a cost. In this study, four MCDM methods, AHP, QFD, FTOPSIS, and PSI, were employed to determine the optimal techniques for dust cleaning in a dry region, Abu Dhabi, UAE. The evaluation considered eight cleaning alternatives across seven criteria, and the data was collected through surveys filled out by experts in the fields of solar and sustainable energy.

The findings of this research highlight the effectiveness of the MCDM methods in ranking the cleaning alternatives. According to the results obtained from AHP, QFD, and PSI, natural cleaning emerged as the top-ranked method, followed either by manual cleaning or surface coatings, as those remained the top 3 best alternatives. However, Fuzzy TOPSIS resulted in a totally different ranking, with acoustic cleaning as the best alternative and natural cleaning as the least favorite. These variations, which could be due to the weighting of the criteria or the use of fuzzy sets instead of abstract real numbers, emphasize the importance of employing multiple evaluation approaches to gain a comprehensive understanding of the optimal cleaning techniques. The sensitivity analysis confirmed the consistency of the decisions made and the optimality of the selected alternatives as the optimal alternatives remained the same, although a −/ + 50% change was applied to the most important criteria.

Although the study suggests that natural cleaning, manual cleaning, and surface coatings are the most favorable methods for maintaining PV panel efficiency, it is recommended that future studies incorporate a larger and more diverse number of experts to further evaluate the cleaning methods and validate the obtained results. Also, including the region’s characteristics to shortlist the alternatives before conducting the analysis could enhance the findings. While the identified optimal cleaning techniques hold value in a general context, the specific needs and conditions of different regions may vary. It is, therefore, crucial to account for regional factors such as climate patterns, environmental conditions, water availability, and local regulations. More importantly, including the impact of each cleaning method on the potential degradation or power loss of the solar PV could be a successful addition, as that would add other considerations to the analysis that would most probably alter the final results. By expanding the scope of research, we can enhance the understanding of PV panel cleaning techniques and contribute to the advancement of sustainable solar energy utilization.

## Data Availability

The datasets used and/or analyzed during the current study are available from the corresponding author upon reasonable request.
